# Limitations of cognitive control on emotional distraction – Congruency in the Color Stroop task does not modulate the Emotional Stroop effect

**DOI:** 10.3758/s13415-021-00935-4

**Published:** 2021-11-04

**Authors:** Elisa Ruth Straub, Constantin Schmidts, Wilfried Kunde, Jinhui Zhang, Andrea Kiesel, David Dignath

**Affiliations:** 1 Department of Psychology, University of Freiburg, Schleichstraße 4, 72076 Tübingen, Germany; 2 Department of Psychology, Cognition, Action, and Sustainability, Engelbergerstr. 41, 79085 Freiburg, Germany

**Keywords:** Cognitive control, Cognitive conflict, Emotional distraction, Emotional Stroop effect, Valence

## Abstract

Emotional information receives prioritized processing over concurrent cognitive processes. This can lead to distraction if emotional information has to be ignored. In the cognitive domain, mechanisms have been described that allow control of (cognitive) distractions. However, whether similar cognitive control mechanisms also can attenuate emotional distraction is an active area of research. This study asked whether cognitive control (triggered in the Color Stroop task) attenuates emotional distraction in the Emotional Stroop task. Theoretical accounts of cognitive control, and the Emotional Stroop task alike, predict such an interaction for tasks that employ the same relevant (e.g., color-naming) and irrelevant (e.g., word-reading) dimension. In an alternating-runs design with Color and Emotional Stroop tasks changing from trial to trial, we analyzed the impact of proactive and reactive cognitive control on Emotional Stroop effects. Four experiments manipulated predictability of congruency and emotional stimuli. Overall, results showed congruency effects in Color Stroop tasks and Emotional Stroop effects. Moreover, we found a spillover of congruency effects and emotional distraction to the other task, indicating that processes specific to one task impacted to the other task. However, Bayesian analyses and a mini-meta-analysis across experiments weigh against the predicted interaction between cognitive control and emotional distraction. The results point out limitations of cognitive control to block off emotional distraction, questioning views that assume a close interaction between cognitive control and emotional processing.

## Introduction

Emotions affect behavior, cognition, and physiology. Negative emotional stimuli, for instance, are considered to have preferential access to awareness and receive prioritized processing over concurrent cognitive processes (Carretié, 2014; Ohman & Mineka, 2001; Yang & Pourtois, 2018). While this is beneficial if emotional information is task-relevant, emotional information that is task-irrelevant interferes with other processes, resulting in impaired task performance (Schmidts et al., 2020; Verbruggen & De Houwer, 2007; Vuilleumier & Huang, 2009). A prominent task to investigate the interference produced by emotional stimuli is the Emotional Stroop task (Watts et al., 1986)*.* This task comprises negative (e.g., “WAR”) and neutral (e.g., “HOUSE”) words written in different print-colors, and participants have to name the print-color of the words. Emotional Stroop effects were expressed in longer latencies and higher error rates when responding to the print-color of negative compared to neutral words (i.e., emotional distraction) and have been quantified in two ways (Frings et al., 2010; McKenna & Sharma, 2004). First, negative valent words impair responses within the current Emotional Stroop trial. Second, the influence of negative words persists in time and impairs performance across trials subsequent to negative word stimuli, even when emotional words are not perceptually present anymore.

The Emotional Stroop paradigm allows researchers to quantify the costs of emotional distraction, which is why it has been widely used as a diagnostic tool in various psychopathologies (e.g., depression, see Mogg & Bradley, 1998; anxiety disorders, see Bar-Haim et al., 2007; social phobia, see Andersson et al., 2006; alcohol, see Lusher et al., 2004; panic disorder, see Harber et al., 2019, for a meta-analysis on fMRI studies on clinical and healthy subjects, see Feng et al., 2018). Current research provides two different theoretical accounts for emotional distraction instigated by Emotional Stroop tasks. According to the attention account (Williams et al., 1996), negative words pull attention towards the irrelevant dimension (i.e., word-processing) which impairs attention for the relevant dimension (i.e., naming the print-color). Alternatively, according to the threat account, negative stimuli have a freezing effect, which slows down all ongoing activities (Algom et al., 2004). For instance, Stolicyn et al. (2017) suggested that emotional words activate the amygdala, which supports representations of task-related stimuli via projections to the medial prefrontal cortex and the orbitofrontal cortex at the expense of other ongoing tasks (Stolicyn et al., 2017).

### Cognitive Control

The Emotional Stroop effect indicates the vulnerability of goal-directed behavior by showing the costs of emotional distraction. However, adaptive human behavior critically relies on the ability to shield current goals from such distraction. Typically, it is assumed that our cognitive system has evolved dedicated control mechanisms that suppress interference from task-irrelevant information (Miller & Cohen, 2001). In the lab, response-interference-tasks (i.e., tasks that activate multiple response options) serve to examine cognitive control processes. A prominent task is the Color Stroop task (Stroop, 1935) in which participants show a delay in reaction times (RTs) and increased error rates when they respond to the print-color (i.e., relevant target dimension) of color words that can be congruent (e.g., the word “BLUE” printed in blue color) or incongruent (e.g., the word “BLUE” printed in red color) to the word’s meaning (i.e., irrelevant distractor dimension). Performance differences between incongruent and congruent trials have been termed congruency effects. Notably, conflict in response-interference-tasks (such as the Color Stroop task) have been found to operate on different timescales (Braver, 2012). Cognitive conflict triggers control in two modes: a reactive control mode that influences the most recent events and permits control during conflict (Scherbaum et al., 2011; Weichart et al., 2020) and a proactive control mode, which operates on a longer timescale following conflict across subsequent trials (Hubbard et al., 2017; Pastötter et al., 2013).

### Interaction of cognitive control and emotion

Is control confined to “cognitive” disturbances or also effective to shield against emotional distraction? Traditionally, cognitive control and emotion have been often described as independent, relying on separate mental faculties (Zajonc, 1980). This view received support from studies showing behavioral (Soutschek & Schubert, 2013) and neural (Egner et al., 2008) evidence for a double dissociation between those tasks that tap into cognitive control and others that tap into emotional processing. More specifically, results from an fMRI study by Egner et al. (2008) revealed that a lateral prefrontal “cognitive control” circuitry that resolved nonemotional conflict can be separated from a rostral anterior cingulate “emotional control” circuitry that resolved emotional conflict (Egner et al., 2008). These findings of a functional segregation were in line with neuroimaging research that showed a separation between the rostral anterior cingulate cortex that is primarily involved in affective processing and regions of the dorsal anterior cingulate cortex and the lateral prefrontal cortex that were associated with nonemotional cognitive processes (Bush et al., 2000). In contrast, other theories question this separation and propose a close interaction between emotion and control (Dignath et al., 2020; Inzlicht et al., 2015; Pessoa, 2008; Shackman et al., 2011; Vermeylen et al., 2020)*.* For instance, Pessoa (2008) suggested that brain regions, such as the amygdala, the orbitofrontal cortex, and the anterior cingulate cortex, function as central hubs that integrate emotional and cognitive information (Pessoa 2008; for a meta-analysis see Shackman et al., 2011). Lesion studies support this view demonstrating that dorsal anterior cingulate cortex lesions cause deficits in the recognition of negative emotions and cognitive response-interference-tasks (Tolomeo et al., 2016). Experimental studies suggest that the anterior cingulate cortex responds similarly to cognitive conflict and negative pictures (Braem et al., 2017). Furthermore, using multivariate pattern classification, Vermeylen et al. (2020) showed overlapping activity for cognitive conflict and negative affect in the medial frontal cortex. Together, neurophysiological studies corroborate the idea that cognitive control and negative affect share a common functional architecture (see also Song et al., 2017).

On a behavioral level, studies show that exerting control can attenuate emotional distraction suggesting that cognitive control may block-off task-irrelevant emotional stimuli (Cohen et al., 2012, 2015; see also Straub et al., 2020). More specifically, Cohen et al. (2012) induced conflict by an arrow flanker task (i.e., participants are required to identify the direction of an arrow which is surrounded by flanking arrows that are either congruent or incongruent with the direction of the arrow in the center) and emotional distraction by negative (vs. neutral) valent pictures. Presenting the response-interference-task and the pictures alternatingly, they found reduced emotional distraction from negative valent picture stimuli in incongruent response-interference-tasks (i.e., reactive control attenuated emotional distraction, see Cohen et al., 2012). In another experiment, the authors used a task-switching design and presented first a response-interference-task that was followed by a color-discrimination task devoid of conflict which measured the impact of a task-irrelevant negative valent picture stimuli presented between both tasks. Results showed that control from incongruent response-interference tasks reduced emotional distraction from negative valent pictures measured in the discrimination task (i.e., proactive control attenuates emotional distraction, see Cohen et al., 2012, 2015, see Goldsmith, 2018 for failure to replicate). However, other studies that addressed the impact of task-irrelevant emotional stimuli on control in response-interference tasks found rather mixed evidence (Fruchtman-Steinbok et al., 2017; Goldsmith, 2018; Hart et al., 2010). This ambiguity of findings is illustrated by Liu et al. (2017) who concluded that “[ …] negative affect has been found to improve, impair, or have no effect on conflict resolution” (Liu et al., 2017, p.69), questioning how generalizable the interplay between cognitive control and emotion is. For instance, Ahmed and Sebastian (2019) suggested that predictability of task conditions (i.e., incongruent or congruent stimuli in conflict tasks, negative or neutral stimuli in emotional tasks) might be a critical moderator for the interaction between cognitive and emotional tasks. They argue that top-down anticipation in cognitive and emotional domains (e.g., when incongruent and congruent as well as negative and neutral stimuli are presented in blocks) is required for conflict-triggered modulation of emotional distraction (Ahmed & Sebastian, 2019).

Against this background, theoretical models are needed that (i) allow a more detailed understanding of a hypothesized interaction of cognitive control and emotion and (ii) thus make testable predictions on how control should modulate emotional distraction. For instance, Cohen et al. (2012, 2015) referred to the conflict monitoring theory to account for their findings that reactive and proactive control reduced emotional distraction (caused by irrelevant negative valent picture stimuli, Cohen et al., 2012, 2015). The conflict monitoring theory (Botvinick et al., 2001) describes control as a feedback loop within a connectionist model. A monitoring unit outputs a measure of competing response activation during a trial, which then scales the activation level of a task demand unit. Increased activation of task demands, representing the current task-set, leads to a change in weightings of related stimulus information and response activation. As a consequence, processing in the next trial is biased towards more relevant information relative to irrelevant information, alleviating further distraction. This model received empirical support from behavioral and neuroimaging studies showing that after incongruent trials, processing of relevant information is enhanced (Egner & Hirsch, 2005) and irrelevant information is suppressed (Stürmer et al., 2002). However, the model as described above, addressed response-interference tasks devoid of emotional stimuli. It remains unclear, therefore, how the original conflict monitoring proposal can account for the empirical findings of Cohen et al. (2012, 2015).

Interestingly, an extension by Wyble et al. (2008) allowed to simulate performance both in the Color Stroop task and in the Emotional Stroop task. Here, emotional distraction is modeled by adding an additional “negative emotional node” that exerts an inhibitory influence on the task demand unit and thereby decreases the activation level of the current task representation. As a consequence, task-irrelevant emotional information impairs performance by reducing cognitive control. Model simulations showed that “an incongruent [Color Stroop] trial reduces the impact of a following negative emotional stimulus by suppressing the emotional node […]” (Wyble et al., 2008, p. 19). Based on the architecture of the model, we derived predictions about how performance in the Emotional Stroop task and the Color Stroop task should interact. Please note that although this prediction is based on simulated data, it corresponds closely to the empirical observation of reduced emotional distraction of pictures after incongruent response-interference-tasks in Cohen’s studies (2012, 2015). Thus, if correct, the model would allow a mechanistic explanation of how cognitive control and emotions might interact in terms of the conflict monitoring theory. However, the tasks used in previous empirical work differ in many aspects from the simulated data in Wyble et al. (2008). For instance, cognitive control measured in Stroop and flanker tasks differs on a behavioral (De Houwer, 2003), physiological (Tillman & Wiens, 2011), and theoretical level (Kornblum et al., 1990; Schuch et al., 2019). Therefore, the goal of the present research is to provide a direct empirical test of the prediction that cognitive control from Color Stroop tasks modulates emotional distraction instigated by Emotional Stroop tasks.

### The present research

We tested whether conflict in the Color Stroop task interacts with emotional distraction in the Emotional Stroop task, as suggested by the model of Wyble et al. (2008). Stimuli in both tasks vary on the same relevant dimension (i.e., naming the print-color in the Color and the Emotional Stroop task). This is important, because it allows to describe the interaction between cognitive control and emotional distraction in terms of the conflict monitoring model. More specifically, in incongruent Color Stroop tasks, attentional weights of the relevant dimension (i.e., naming the print-color) are increased and conflict from Color Stroop tasks should facilitate print-color-naming in Emotional Stroop tasks. Accordingly, proactive and reactive cognitive control from incongruent trials in the Color Stroop tasks should decrease emotional distraction instigated by the Emotional Stroop task. However, in congruent Color Stroop stimuli, attention is directed towards the irrelevant word-meaning *and* the relevant print-color (because both predict the correct response) and print-color-naming in Emotional Stroop tasks is not facilitated. These considerations are in line with empirical evidence of studies showing that cognitive control generalizes across cognitive tasks that share the same relevant dimension (Kunde & Wühr, 2006; Notebaert & Verguts, 2008). Furthermore, the two tasks used in our design share not only the same relevant dimension but also the irrelevant stimulus dimension (i.e., ignoring the semantic meaning of the carrier word in the Color Stroop and the Emotional Stroop task). Critically, tasks differ according to the response set associated with the irrelevant dimension. While the Color Stroop task’s irrelevant dimension affords a response that either matches or mismatches the correct response afforded by the relevant stimulus dimension, this is not true for the Emotional Stroop task. Naming the color of a negative valent or neutral word (i.e., irrelevant stimulus dimension) is not mapped to any response option and thus it can neither be congruent nor incongruent with color-naming (e.g., the word “ILLNESS” is not mapped to any distinct print-color). Based on this, emotional distraction instigated by Emotional Stroop tasks and congruency effects from Color Stroop tasks can be considered as two functional distinct effects (i.e., a threat induced slow-down in the Emotional Stroop task versus competition between incongruent stimuli in the Color Stroop task, see Algom et al., 2004).

### Hypothesis

**Fig. 1:**
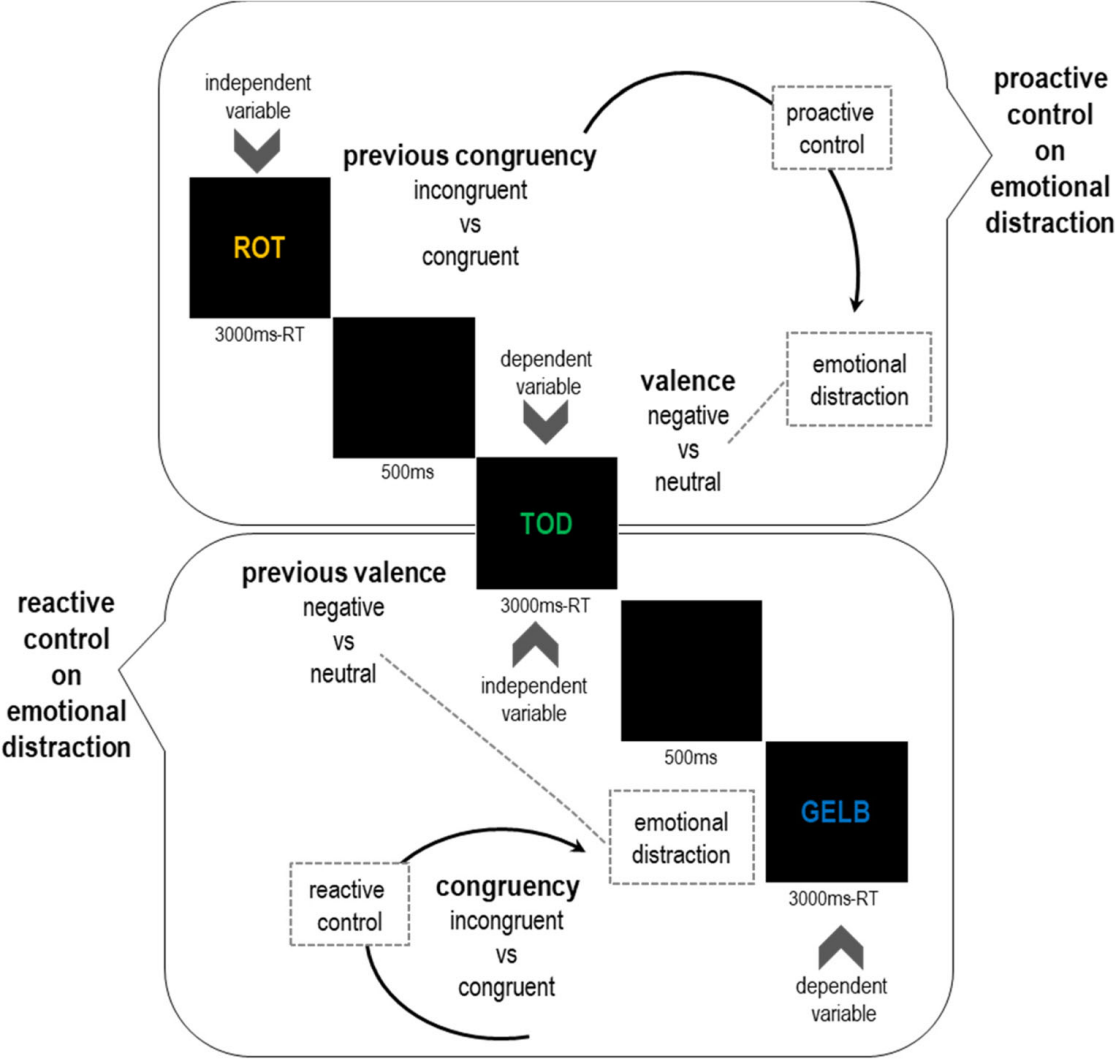
Proactive and Reactive Control on emotional distraction. *Note*. Example trial sequence with Color Stroop stimuli and Emotional Stroop stimuli. In 'proactive control on emotional distraction', control from the Color Stroop task modulates emotional distraction in the subsequent Emotional Stroop task and in 'reactive control on emotional distraction' control from the Color Stroop task modulates emotional distraction from the previous Emotional Stroop task.

To probe the hypothesized interaction between cognitive control and emotional distraction, we intermixed Color and Emotional Stroop tasks. On each trial, participants had to name the print-color of a colored word (same relevant dimension in both tasks). To create Color Stroop and Emotional Stroop conditions, the type of colored words alternated on each trial between color words and negative/neutral valent words (both tasks vary on the same irrelevant dimension). Critically, while negative/neutral valent words were not related to any print-color, all color words presented referred to the same set of colors as used for print-color (different irrelevant response set between both tasks). Color Stroop trials, but not Emotional Stroop trials, created congruent (e.g., “RED” printed in red) and incongruent (e.g., “RED” printed in green) conditions that allowed to express performance as a congruency effect (incongruent – congruent trials). In reverse, Emotional Stroop trials, but not Color Stroop trials, created neutral (e.g., “HOUSE” printed in red) and negative valent (e.g., “WAR” printed in red) conditions that allowed to express performance as an Emotional Stroop effect (negative – neutral trials). This alternating-runs design (i.e., Color Stroop task - Emotional Stroop task - Color Stroop task - Emotional Stroop task…) allowed us to test whether emotional distraction is modulated by both, proactive and reactive control. As explained above, emotional distraction occurs in the Emotional Stroop task and also persists in time and occurs in the task subsequent to the negative word stimuli and thus we can measure the emotional distraction at two different times. Accordingly, this design tested (i) how proactive control from the Color Stroop task modulates emotional distraction in the subsequent Emotional Stroop task ('proactive control on emotional distraction') and (ii) how reactive cognitive control from the Color Stroop task modulates emotional distraction that persists in time and stems from the previous Emotional Stroop task ('reactive control on emotional distraction'). We expected that cognitive control reduces emotional distraction and thus hypothesized that (i) proactive cognitive control from Color Stroop tasks reduces emotional distraction in subsequent Emotional Stroop tasks and (ii) that reactive cognitive control reduces emotional distraction instigated by the previous Emotional Stroop task (Figure 1).

In terms of predictability (Ahmed & Sebastian, 2019), we presented congruency (congruent vs. incongruent stimuli in Color Stroop tasks) and valence (negative vs. neutral stimuli in Emotional Stroop tasks) conditions in different configurations across the Experiments (i.e., Experiment 1a & Experiment 1b blocked valence and congruency, Experiment 2 manipulated congruency trialwise, Experiment 3 manipulated valence trialwise).

## Experiment 1a

In Experiment 1a, congruency in Color Stroop and valence in Emotional Stroop tasks was manipulated blockwise, which predicts the largest Emotional Stroop effects (McKenna & Sharma, 2004; Phaf & Kan, 2007). We aimed to find proactive and reactive control on emotional distraction.

### Methods

Previous research that investigates how congruency effects generalize across different tasks that share the same relevant dimension observed effect sizes ranging from *dz* = 0.956 (Notebart & Verguts, 2008) to *dz* = 2.11 (see Kunde & Wühr, 2006, Experiment 1). Power analyses using G*Power suggested a minimum sample size of N = 14 to detect an effect of *dz* = 0.956 (with α = 0.05 and 1-β = 0.9) for the within-groups comparison between emotional distraction in congruent and incongruent conditions. The present study tested 40 participants and was completed at the University of Freiburg, Germany. All participants reported normal or corrected-to-normal vision and were compensated with either course credit or money. Exclusion criteria were identical for all experiments and defined a priori based on conventions of our workgroup. We excluded participants with (i) random responses (error rate above 50%) and (ii) with an error rate above three standard deviations (*SD*s) from the remaining sample after excluding random responses. In Experiment 1a, no participant was excluded due to random responses. Data of one participant were excluded due to error rates above three standard deviations (*SD*s). Hence, we analyzed data of 39 participants (1 left-handed, 27 female, *M*age = 26.6 years).

#### Apparatus, Procedure, and Stimuli

The experiment was programmed and presented with e-Prime software 2.0, E-Studio (version: 2.0.10.252; Schneider et al., 2002). Responses were collected with standard German QWERTZ-keyboard. After providing informed consent, participants were instructed to respond to the four different print-colors in which words on the screen were presented via four previously assigned keys (i.e., green, red, yellow, blue). Color-to-key mapping was counterbalanced across participants. Each trial started with a Color Stroop task (i.e., a color word (“RED,” “GREEN,” “BLUE,” “YELLOW”) presented in either green (RGB = 0, 128, 64), red (255, 0, 0), blue (0, 255, 255), or yellow (255, 255, 0) print-color). Participants had to classify the print-color (target) and ignore the word-meaning of the carrier word (distractor). Stimuli were either congruent to the word-meaning (e.g., “GREEN” written in green print-color) or incongruent (e.g., “GREEN” written in yellow print-color). For incongruent stimuli, all possible color-word combinations were used (e.g., the word “BLUE” can be printed in red, yellow, or green color). In each trial, a Color Stroop task was followed by an Emotional Stroop task (i.e., a word with either negative (distractor) or neutral valence presented in green, red, yellow, or blue print-color (target). Participants had to respond to the print-color with the previously assigned keys. Stimuli comprised 20 neutral and 20 negative words taken from the Berlin Affective Word List (BAWL, Võ et al., 2009). According to the database, mean arousal was rated on a 6-point scale ranging from 0 to 5 (0 = not arousing to 5 = highly arousing) and valence was rated on a 7-point scale ranging from −3 (very negative) through 0 (neutral) to 3 (very positive). Ratings of the arousal of stimuli used in our experiment corresponded to mean ratings of 4.25 (*SD* = 0.32) in negative stimuli and 1.76 (*SD* = 0.09) in neutral stimuli, *t*(38) = 33.128, *p* < 0.01, and ratings of the valence of stimuli correspond to mean ratings of −2.66 (*SD* = 0.17) in negative stimuli and 0.03 (*SD* = 0.11) in neutral stimuli, *t*(38) = −59.290, *p* < 0.01 , respectively. We matched the number of letters in negative and neutral words but not word frequency of both conditions (i.e., in our stimulus set, frequency of use is lower in negative compared to neutral words), which have been shown to increase Emotional Stroop effects (Kahan & Hely, 2008; Larsen et al., 2006).

All words were written in capital letters and one letter subtended 1.72° (width) × 1.33° (height) of visual angle, measured from a viewing distance of 60 cm. In Experiment 1a, both congruency (congruent or incongruent) of the Color Stroop task and valence (negative or neutral) of the word stimuli in the Emotional Stroop task were manipulated blockwise resulting in four block conditions (i.e., (i) incongruent color-word stimuli and negative word stimuli, (ii) congruent color word stimuli and negative word stimuli, (iii) incongruent color-word stimuli and neutral word stimuli, and (iv) congruent color word stimuli and neutral word stimuli). Each block was presented 3 times, resulting in 12 blocks in total. Within these 12 blocks, the 4 different block conditions were presented in random order. Each block comprised 20 trials in which each negative or neutral word was presented once, resulting in 240 trials in total. Stimuli within one block were presented in random order. Participants made self-paced rests after each block. A trial started with the presentation of a word stimulus that remained on the screen for 3,000 ms or until a response was registered, followed by a 500-ms Inter-Trial-Interval. A trial sequence was identical for the Color and the Emotional Stroop task. Participants received accuracy feedback after each task and were asked to respond as quickly and accurately as possible. They started with a practice session, including accuracy feedback of 24 trials. After finishing the Experiment participants completed a German version of the Emotion Regulation Questionnaire (ERQ) (Gross & John, 2012; Loch et al., 2011) and a German version of Trait Anxiety Inventory (STAI-T) (Laux, Glanzmann et al., 1981; Spielberger & Sydeman, 1994).

#### Data Analysis

Mean RTs and error rates were calculated separately for Color and Emotional Stroop tasks. Data were analyzed with a repeated-measures Analysis of Variance (ANOVA) with the within-subject factors *congruency* (incongruent vs congruent) and *valence* (negative vs. neutral). Testing 'proactive control on emotional distraction´, independent variables were previous congruency (*pre-congruency*) of Color Stroop tasks (i.e., congruent or incongruent) and valence (*valence*) of Emotional Stroop tasks (i.e., negative or neutral). RTs and error rates in Emotional Stroop tasks served as dependent variables. Testing 'reactive control on emotional distraction', independent variables were previous valence (*pre-valence*) of Emotional Stroop tasks and congruency (*congruency*) of Color Stroop tasks. RTs and error rates in Color Stroop tasks served as dependent variables.

Participant’s trait anxiety (STAI-T score) and the emotion regulation strategies (ERQ-Score) were correlated with modulation of emotional distraction by cognitive control. Bonferroni corrected *p*-values were calculated via dividing the desired alpha level (i.e., α = 0.05) by the number of comparisons (i.e., ERQ and 'proactive control on emotional distraction', ERQ and 'reactive control on emotional distraction', STAI-T and 'proactive control on emotional distraction', STAI-T and 'reactive control on emotional distraction'; n = 4), resulting in a least significant difference *p*-value of 0.05/4 = 0.0125.

Relevant null-effects (i.e., nonsignificant interaction effects between *congruency* and *valence*) were further analyzed by calculating Bayes Factors (BF) using JASP (Jarosz & Wiley, 2014). The Bayesian approach is a model selection procedure that compares the likelihood of the data considered under both, the null- and the alternative-hypothesis via calculation of the BF01. The BF01 gives an index of how strong data are in favor of the null-hypothesis. The default setting of JASP Bayesian statistics paired *t*-test was used as prior, which consists of a Cauchy distribution (i.e., a t-distribution with a single degree of freedom) with its parameter set to *r* = 0.707.

## Results

Trials in which participants committed an error in the Color or the Emotional Stroop task and all trials following an error trial were excluded (5.8% and 4.3% of responses in the Color and the Emotional Stroop task, respectively). Furthermore, RTs that deviate more than three *SDs* for each participant and each condition (i.e., each cell of the ANOVA design) were removed from RT analyses (1.4% and 1.8% of responses in the Color and the Emotional Stroop task, respectively).

### Reaction times

#### Impact of proactive cognitive control on emotional distraction

Results of the two-way ANOVA with the within-subject factors *pre-congruency* and *valence* and performance in the Emotional Stroop task serving as dependent variable revealed no significant main effects of *pre-congruency* and *valence* and no interaction of *pre-congruency* × *valence*, (*Fs* < 1*).*

#### Impact of reactive cognitive control on emotion distraction

Results of the two-way ANOVA with *pre-valence* and *congruency* as within-subject factors and performance in the Color Stroop task as dependent variable revealed a significant main effect of *pre-valence F*(1,38) = 5.919, *p* = 0.020, ${\eta}_p^2$= 0.135, indicating prolonged RTs in Color Stroop tasks that were preceded by negative word stimuli (*M* = 760 ms, *SE* = 25 ms) compared with RTs in Color Stroop tasks that were preceded by neutral word stimuli (*M* = 740 ms, *SE* = 25 ms), a significant main effect of *congruency*, *F*(1,38) = 85.177, *p* < 0.001, ${\eta}_p^2$= 0.692 demonstrating faster responses in congruent Color Stroop tasks (*M* = 683 ms, *SE* = 22 ms) compared with incongruent Color Stroop tasks (*M* = 817 ms, *SE* = 29 ms) but no interaction of *pre-valence* × *congruency* (*F < 1).*

### Bayesian Analysis

Quantification of the results by BFs assumes that in 'proactive control on emotional distraction', the null-hypothesis indicates that emotional distraction within Emotional Stroop tasks preceded by incongruent Color Stroop tasks is not smaller compared with emotional distraction within Emotional Stroop tasks preceded by congruent Color Stroop tasks. The alternative-hypothesis indicates that emotional distraction within Emotional Stroop tasks preceded by incongruent Color Stroop tasks is smaller compared with emotional distraction within Emotional Stroop tasks preceded by congruent Color Stroop tasks. The corresponding BF provides positive evidence for the null-hypothesis relative to the alternative-hypothesis (BF01 = 3.194) and indicates that the data are three times more likely under the null-hypothesis than under the alternative-hypothesis (Schönbrodt & Wagenmakers, 2018). In the analysis of 'reactive control on emotional distraction', the null-hypothesis indicates that emotional distraction in Color Stroop tasks instigated by preceding negative Emotional Stroop tasks is not smaller in incongruent compared with congruent Color Stroop tasks. The alternative-hypothesis indicates that emotional distraction in Color Stroop tasks instigated by preceding negative Emotional Stroop tasks is smaller in incongruent compared with congruent Color Stroop tasks. The corresponding BF01 provides positive evidence for the null-hypothesis relative to the alternative-hypothesis (BF01 = 7.086) and indicates that the data are seven times more likely under the null-hypothesis than under the alternative-hypothesis.

### Error Rates

Analogous analyses were performed on error rates.

#### Impact of proactive cognitive control on emotional distraction

Results of the two-way ANOVA with *pre-congruency* and *valence* serving as within-subject factors and performance in the Emotional Stroop task serving as dependent variable revealed no significant main effect of *valence, F*(1,38) = 1.779, *p* = 0.190*,* ${\eta}_p^2$= 0.045. The main effect of *pre-congruency* and the interaction effect of *pre-congruency* and *valence* were not significant (Fs *<* 1).

#### Impact of reactive cognitive control on emotional distraction

Results of the two-way ANOVA with *pre-valence* and *congruency* as within-subject factors and performance in the Color Stroop task serving as dependent variable revealed no significant main effects of *pre-valence* and *congruency* (*Fs <* 1), but a significant interaction effect, *F*(1,38) = 6.669, *p* = 0.014, ${\eta}_p^2$= 0.149 with more emotional distraction in congruent Color Stroop tasks (*M* = 8.5%, *SE* = 3.7%) compared with incongruent Color Stroop tasks (*M* = −7.7%, *SE* = 4.2%).

### Bayesian Analysis

The corresponding Bayesian Analysis indicates that in 'proactive control on emotional distraction' data are seven times more likely under the null-hypothesis than under the alternative-hypothesis (BF01 = 6.769). BFs were calculated for non-significant interaction effects only.

### Questionnaires

Correlations between ERQ- and STAI-Scores^1^ and modulation of emotional distraction were not significant^2^ ('proactive control on emotional distraction', ERQ: *r*(38) = −0.030, *p* = 0.858, STAI-T: *r*(39) = 0.200, *p* = 0.222, 'reactive control on emotional distraction', ERQ: *r*(38) = 0.186, *p* = 0.264, STAI-T: *r*(39) = 0. 097, *p* = 0.561).

## Experiment 1b

We aimed to boost interference effects from emotional stimuli to further analyze the modulation of emotional distraction by cognitive control. Therefore, we changed the duration and inter-trial-intervals of stimuli. The blank time between tasks was set to 6.9 ms^3^ and the duration of stimuli presentation was reduced to 2000 ms. This modification was based on studies by McKenna (1986) and McKenna & Sharma (1995), who observed reliable interference effects in Emotional Stroop tasks under time pressure (McKenna, 1986; McKenna & Sharma, 1995). We used the same stimuli and procedure as in Experiment 1a but added four word stimuli from the BAWL database to the negative and the neutral word categories, resulting in 24 trials per block. Each block was presented five times, resulting in 20 blocks and 480 trials in total. We added the constraint that the four block conditions (i.e., (i) incongruent Color Stroop stimuli and negative words, (ii) congruent Color Stroop stimuli and negative words, (iii) incongruent Color Stroop stimuli, and neutral words (iv) congruent Color Stroop stimuli and neutral words) did not repeat throughout the experiment. Furthermore, stimuli within one block were presented randomly with the constraint that the task-relevant dimension (i.e., print-color of the words) did not repeat in two consecutive trials. In the practice session, we presented 100 words of randomly mixed letters in the four colors and accuracy feedback was provided so that participants learned the color-to-key mapping.

### Methods

#### Participants

Study site, results of the power analyses, number of recruited participants, as well as inclusion and exclusion criteria for participants were the same as in Experiment 1a. No participant was excluded due to random responses. Data of one participant was excluded due to error rates of three *SD*s above the mean error rates. Hence, we analyzed data of 39 participants (5 left-handed, 30 female, *M*age = 24.70 years).

### Results

Trials in which participants committed an error in the Color or the Emotional Stroop task and all trials following an error trial were excluded (8.9% and 8.4% of responses in the Color and the Emotional Stroop task, respectively). Furthermore, RTs that deviate more than three *SDs* for each participant and each condition (i.e., each cell of the ANOVA design) were removed from RT analyses (1.2% and 1.3% of responses in the Color and the Emotional Stroop task, respectively).

#### Reaction times

#### Impact of proactive cognitive control on emotional distraction

Results of the two-way ANOVA with the within-subject factors *pre-congruency* and *valence* and performance in the Emotional Stroop task serving as dependent variable revealed a significant main effect of *pre-congruency, F*(1,38) = 6.268, *p* = 0.017*,* ${\eta}_p^2$= 0.142, demonstrating faster responses after congruent Color Stroop tasks (*M* = 876 ms*, SE* = 17 ms) compared with incongruent Color Stroop tasks *(M* = 860 ms, *SE* = 15 ms) and *valence, F*(1,38) = 9.462, *p* = 0.004*,* ${\eta}_p^2$= 0.199, indicating emotional distraction demonstrated in prolonged RTs in negative Emotional Stroop tasks (*M* = 877 ms*, SE* = 16 ms) compared with neutral Emotional Stroop tasks *(M* = 859 ms*, SE* = 15 ms) but no interaction of *pre-congruency* × *valence*, *F*(1,38) = 0.001, *p* = 0.973*,* ${\eta}_p^2$ < 0.001.

#### Impact of reactive cognitive control on emotional distraction

Results of the two-way ANOVA with the within-subject factors *pre-valence* and *congruency* and performance in the Color Stroop task serving as dependent variable revealed a significant main effect of *pre-valence*, *F*(1,38) = 5.132, *p* = 0.029*,* ${\eta}_p^2$= 0.119, indicating emotional distraction demonstrated in prolonged RTs in Color Stroop tasks that were preceded by negative word stimuli (*M* = 875 ms, *SE* = 17 ms) compared with RTs in Color Stroop tasks that were preceded by neutral word stimuli (*M* = 860 ms, *SE* = 16 ms). Furthermore, there was a significant main effect of *congruency*, *F*(1,38) = 150.488, *p* < 0.001*,* ${\eta}_p^2$= 0.798 demonstrating faster responses in congruent (*M* = 806 ms, *SE* = 15 ms) compared with incongruent Color Stroop tasks *(M* = 929 ms*, SE* = 20 ms*)*, but no interaction of *pre-valence* × *congruency, F*(1,38) = 0.149, *p* = 0.702*,* ${\eta}_p^2$= 0.004.

#### Bayesian Analysis

The corresponding Bayesian Analysis indicates that in 'proactive control on emotional distraction', data are six times more likely under the null-hypothesis than under the alternative-hypothesis (BF01 = 5.948). In 'reactive control on emotional distraction', data are four times more likely under the null-hypothesis than under the alternative-hypothesis (BF01 = 4.189).

#### Error Rates

Analogous analyses were performed on error rates.

#### Impact of proactive cognitive control on emotional distraction

Results of the two-way ANOVA with the within-subject factors *pre-congruency* and *valence* and performance in the Emotional Stroop task serving as dependent variable revealed no significant main and interaction effects (all *Fs < 1*).

#### Impact of reactive cognitive control on emotional distraction

Results of the two-way ANOVA with the within-subject factors *pre-valence* and *congruency* and performance in the Color Stroop task serving as dependent variable revealed no significant main effect of *pre-valence, F*(38) = 1.566*, p* = 0.218 *,* ${\eta}_p^2$= 0.040, but a significant main effect of *congruency, F*(38) = 13.827*, p* = 0.001, ${\eta}_p^2$= 0.267, demonstrating less errors in congruent tasks (*M* = 6.6%, *SE* = 0%) compared to incongruent tasks (*M* = 8.7%, *SE* = 1%). Interaction effects between *pre-valence* and *congruency* were not significant (*F <* 1).

#### Bayesian Analysis

The corresponding Bayesian Analysis indicates that in 'proactive control on emotional distraction', data are eight times more likely under the null-hypothesis than under the alternative-hypothesis (BF01 = 8.133). In 'reactive control on emotional distraction', data are seven times more likely under the null-hypothesis than under the alternative-hypothesis (BF01 = 6.860).

#### Questionnaires

Correlations between ERQ- and STAI-T scores and modulation of emotional distraction were not significant ('proactive control on emotional distraction', ERQ: *r*(39) = 0.017, *p* = 0.919, STAI-T: *r*(39) = −0.392, *p* = 0.014^4^, 'reactive control on emotional distraction', ERQ: *r*(39) = 0.013, *p* = 0.938, STAI-T: *r*(39) = −0.070, *p* = 0.674).

## Experiment 2

Modifications in the design of Experiment 1a revealed emotional distraction within and subsequent to the Emotional Stroop tasks in Experiment 1b. However, contrary to our predictions, we did not find any evidence for 'proactive control on emotional distraction' or 'reactive control on emotional distraction'. We consider two possible reasons for the null-effects that implicate further manipulations in the following experiments. First, we suggest that *predictability of congruency* (i.e., conflict tasks were presented in blocks with either congruent or incongruent stimuli in Experiments 1a and 1b) may play a role in the activation of top-down anticipatory control mechanism (Ahmed & Sebastian, 2019; Grimshaw et al., 2018). We removed the predictability of the Color Stroop task’s congruency in Experiment 2 by manipulating congruency trialwise. Procedure and Stimuli were the same as in Experiment 1b. In each block incongruent (50%) and congruent (50%) Color Stroop stimuli were presented in random order and valence of the Emotional Stroop stimuli was either negative or neutral within one block. There were 20 blocks in total, each comprising 24 trials. The two different block conditions (i.e., (i) incongruent and congruent Color stimuli with negative words, and (ii) incongruent and congruent Color stimuli with neutral words) were presented in random order.

### Methods

#### Participants

The study was completed at the University of Würzburg, Germany. Power analyses, inclusion, and exclusion criteria for participants were the same as in Experiments 1a and 1b. A total of 41 participants completed the study. No participants were excluded due to random answering or error rates of 3 *SDs* above the mean error rates. Hence, we analyzed data of 41 participants (3 left-handed, 28 females, *M*age = 24.37 years).

### Results

Trials in which participants committed an error in the Color or the Emotional Stroop task and all trials following an error trial were excluded (8.9% and 8.1% of responses in the Color and the Emotional Stroop task, respectively). Furthermore, RTs that deviate more than three *SDs* for each participant and each condition (i.e., each cell of the ANOVA design) were removed from RT analyses (1.0% and 1.3% of responses in the Color Stroop task and the Emotional Stroop task, respectively).

#### Reaction times

#### Impact of proactive cognitive control on emotional distraction

Results of the two-way ANOVA with the within-subject factors *pre-congruency* and *valence* and performance in the Emotional Stroop task serving as dependent variable revealed significant main effects of *pre-congruency, F*(1,40) = 4.103, *p* = 0.050*,* ${\eta}_p^2$= 0.093, demonstrating faster responses after congruent (*M* = 864 ms*,* *SE* = 16 ms) compared with incongruent Color Stroop tasks *(M* = 878 ms, *SE* = 16 ms) and a *valence, F*(1,40) = 9.860, *p* = 0.003*,* ${\eta}_p^2$= 0.198, indicating emotional distraction demonstrated in prolonged RTs in negative (*M* = 878 ms , *SE* = 16 ms) compared with neutral Emotional Stroop tasks *(M* = 864 ms*,* *SE* = 16 ms) but no interaction of *pre-congruency* × *valence*, *F*(1,40) = 0.027, *p* = 0.871*,* ${\eta}_p^2$ < 0.001.

#### Impact of reactive cognitive control on emotional distraction

Results of the two-way ANOVA with the within-subject factors *pre-valence* and *congruency* and performance in the Color Stroop task serving as dependent variable revealed significant main effects of *pre-valence F*(1,40) = 11.246, *p* = 0.002*,* ${\eta}_p^2$= 0.219, indicating emotional distraction demonstrated in prolonged RTs in Color Stroop tasks that were preceded by negative Emotional Stroop tasks (*M* = 893 ms, *SE* = 17 ms) compared with RTs in Color Stroop tasks that were preceded by neutral word stimuli (*M* = 873 ms, *SE* = 15 ms) and *congruency*, *F*(1,40) = 236.559, *p* < 0.001*,* ${\eta}_p^2$= 0.855, demonstrating faster responses in congruent (*M* = 818 ms, *SE* = 16 ms) compared with incongruent Color Stroop tasks *(M* = 948 ms*, SE* = 17 ms), but no interaction of *pre-valence* × *congruency, F*(1,40) = 3.214 , *p* = 0.081*,* ${\eta}_p^2$= 0.074. Emotional distraction was descriptively larger in incongruent Color Stroop tasks (*M* = 28 ms, *SE* = 8 ms) compared with congruent Color Stroop tasks (*M* = 13 ms, *SE* = 8 ms), which is against the prediction (see also Bayesian Analysis below).

#### Bayesian Analysis

The corresponding Bayesian Analysis indicates that in 'proactive control on emotional distraction', data are five times more likely under the null-hypothesis than under the alternative-hypothesis (BF01 = 5.204). In 'reactive control on emotional distraction', data are 15 times more likely under the null-hypothesis than under the alternative-hypothesis (BF01 = 15.357).

#### Error Rates

Analogous analyses were performed on error rates.

#### Impact of proactive cognitive control on emotional distraction

Results of the two-way ANOVA with the within-subject factors *pre-congruency* and *valence* and performance in the Emotional Stroop task serving as dependent variable revealed no significant main and interaction effects (all *Fs* < 1).

#### Impact of reactive cognitive control on emotional distraction

Results of the two-way ANOVA with the within-subject factors *pre-valence* and *congruency* and performance in the Color Stroop task serving as dependent variable revealed a significant main effect of *pre-valence, F*(1,40) = 6.090, *p* = 0.018*,* ${\eta}_p^2$= 0.132, indicating emotional distraction demonstrated in more errors in Color Stroop tasks that were preceded by negative Emotional Stroop tasks (*M* = 8.4%, *SE* = 1%) compared with Color Stroop tasks that were preceded by neutral Emotional Stroop tasks *(M* = 7.3%*, SE* = 1%). Furthermore, there was a significant main effect of *congruency, F*(1,40) = 20.828, *p* < 0.001, ${\eta}_p^2$= 0.342, demonstrating less errors in congruent *(M* = 6.5%, *SE* = 1) compared with incongruent Color Stroop tasks (*M* = 9.2%, *SE* = 1%) and an interaction of *pre-valence* × *congruency*, *F*(1,40) = 5.376, *p* = 0.026*,* ${\eta}_p^2$ = 0.118 with more emotional distraction in incongruent Color Stroop tasks (*M* = 2.1%, *SE* = 0.7%) compared with congruent Color Stroop tasks (*M* = 0.1%, *SE* = 0.5%), which is against the expected results.

#### Bayesian Analysis

The corresponding Bayesian Analysis indicates that in 'proactive control on emotional distraction', data are ten times more likely under the null-hypothesis than under the alternative-hypothesis (BF01 = 10.483).

#### Questionnaires

Correlations between the questionnaires and modulation of emotional distraction by congruency were not significant ('proactive control on emotional distraction', ERQ: *r*(41) = 0.237, *p* = 0.136. STAI-T: *r*(41) = 0.016, *p* = 0.921, 'reactive control on emotional distraction', ERQ: *r*(41) = −0.161, *p* = 0.316, STAI-T: *r*(41) = 0.126 , *p* = 0.431).

## Experiment 3

In Experiments 1b and 2, we found emotional distraction within and subsequent to the Emotional Stroop tasks but no evidence for 'proactive control on emotional distraction' or 'reactive control on emotional distraction'. We consider *predictability of valence* (i.e., Emotional Stroop tasks were presented in blocks with either negative or neutral valent stimuli in all previous experiments) as a second possible reason for the null-effects. Hence, we removed the predictability of valence in Emotional Stroop tasks. The procedure and stimuli used in Experiment 3 were the same as in Experiment 2 with the exception that the valence in Emotional Stroop tasks was manipulated trialwise. We found no differences in the size of congruency effects between blocked or trialwise presentation of Color Stroop stimuli (Experiment 1b blockwise, *M*_Congruencyeffect_ = 125.77, SD = 63.36, Experiment 2*M*_Congruencyeffect_ = 129.25, SD = 54.87, *t*(80) = −0.263, *p* = 0.793) and thus presented Color Stroop stimuli trialwise due to larger congruency effects in Experiment 2 compared with Experiment 1b. Within each block, congruency of the Color Stroop task and valence of the Emotional Stroop task were presented randomly. There were 14 blocks in total; each included 48 trials. We included four catch trials in each block (i.e., two randomly chosen words of both, the negative and the neutral categories printed in gray) and instead of indicating the word’s print-color, participants had to categorize the meaning of these words into “negative” or “neutral” via keypress.

### Methods

#### Participants

The study was completed at the University of Freiburg. Results of the Power analyses and inclusion criteria for participants were the same as in Experiments 1a, b, and 2. Participants were excluded if the error rate in the categorization of the catch trial word’s meaning exceeded 50%. A total of 42 participants completed the study. No participants were excluded due to random answering, one participant was excluded due to error rates of three *SD*s above the mean error rates, and one participant was excluded due to an error rate above 50% in the catch trials. Hence, we analyzed data of 40 participants (4 left-handed, 25 females, *M*age = 27.73 years).

### Results

Trials in which participants committed an error in the Color or the Emotional Stroop task and all trials following an error trial were excluded (9.5% and 9.1% of responses in the Color Stroop task and the Emotional Stroop task, respectively). Furthermore, RTs that deviate more than three SDs for each participant and each condition (i.e., each cell of the ANOVA design) were removed from RT analyses (0.9% and 1.1% of responses in the Color Stroop task and the Emotional Stroop task, respectively).

#### Reaction times

#### Impact of proactive cognitive control on emotional distraction

Results of the two-way ANOVA with the within-subject factors *pre-congruency* and *valence* and performance in the Emotional Stroop task serving as dependent variable revealed a significant main effect of *pre-congruency, F*(1,39) = 11.378, *p* = 0.002*,* ${\eta}_p^2$= 0.226, demonstrating faster responses after congruent (*M* = 873 ms*, SE* = 18 ms) compared with incongruent Color Stroop tasks (*M* = 890 ms, *SE* = 17 ms)*.* Results revealed no significant main effect of *valence*, *F*(1,39) = 0.158, *p* = 0.693*,* ${\eta}_p^2$= 0.004 and no significant interaction of *pre-congruency* × *valence*, *F*(1,39) = 0.787, *p* = 0.381*,* ${\eta}_p^2$ = 0.020.

#### Impact of reactive cognitive control on emotional distraction

Results of the two-way ANOVA with the within-subject factors *pre-valence* and *congruency* and performance in the Color Stroop task serving as dependent variable revealed a significant main effect of *pre-valence, F*(1,39) = 13.138, *p* = 0.001*,* ${\eta}_p^2$= 0.252 indicating emotional distraction demonstrated in prolonged RTs in Color Stroop tasks that were preceded by negative Emotional Stroop tasks (*M* = 901 ms, *SE* = 18 ms) compared with RTs in Color Stroop tasks that were preceded by neutral word stimuli (*M* = 887 ms, *SE* = 18 ms), a significant main effect of *congruency*, *F*(1,39) = 216.185, *p* < 0.001*,* ${\eta}_p^2$= 0.847 demonstrating faster responses in congruent (*M* = 831 ms*, SE* = 18 ms) compared with incongruent Color Stroop tasks *(M* = 957 ms, *SE* = 19 ms), but no interaction of *pre-valence* × *congruency, F*(1,39) = 0.179 , *p* = 0.674*,* ${\eta}_p^2$= 0.005.

#### Bayesian Analysis

The corresponding Bayesian Analysis indicates that in 'proactive control on emotional distraction', data are three times more likely under the null-hypothesis than under the alternative-hypothesis (BF01 = 2.530) and in 'reactive control on emotional distraction', data are eight times more likely under the null-hypothesis than under the alternative-hypothesis (BF01 = 7.883).

#### Error Rates

Analogous analyses were performed on error rates.

#### Impact of proactive cognitive control on emotional distraction

Results of the two-way ANOVA with the within-subject factors *pre-congruency* and *valence* and performance in the Emotional Stroop task serving as the dependent variable revealed no significant main or interaction effects (*Fs* < 1).

#### Impact of reactive cognitive control on emotional distraction

Results of the two-way ANOVA with the within-subject factors *pre-valence* and *congruency* and performance in the Color Stroop task serving as dependent variable revealed no significant main effect of *pre-valence, F*(1,39) = 4.071, *p* = 0.051*,* ${\eta}_p^2$= 0.095. Descriptively, error rates in Color Stroop tasks that were preceded by negative Emotional Stroop tasks were higher (*M* = 7.9%, *SE* = 1%) compared with Color Stroop tasks that were preceded by neutral Emotional Stroop tasks (*M* = 7.4%*, SE* = 1%). Furthermore, there was a significant main effect of *congruency, F*(1,39) = 14.709, *p* < 0.001, ${\eta}_p^2$= 0.274*,* demonstrating less errors in congruent (*M* = 6.5%, *SE* = 1%) compared with incongruent Color Stroop tasks (*M* = 8.8%, *SE* = 1%) but no significant interaction of *pre-valence* × *congruency* (*F < 1)*.

#### Bayesian Analysis

The corresponding Bayesian Analysis indicates that in 'proactive control on emotional distraction', data are five times more likely under the null-hypothesis than under the alternative-hypothesis (BF01 = 5.180) and in 'reactive control on emotional distraction', data are four times more likely under the null-hypothesis than under the alternative-hypothesis (BF01 = 3.527).

#### Questionnaires

Correlations between ERQ- und STAI-T-scores and modulation of emotional distraction by congruency were not significant ('proactive control on emotional distraction', ERQ: *r*(40) = −0.244 , *p* = 0.129, STAI-T: *r*(40) = −0.175, *p* = 0.279, 'reactive control on emotional distraction', ERQ: *r*(40) = −0.012, *p* = 0.944, STAI-T: *r*(40) = 0.220, *p* = 0.172).

### Mini-Meta-Analysis

We subjected the four experiments to a meta-analytic summary to get an overall estimate of the influences of cognitive control on emotional distraction. We wanted to attain an average true effect in the set of our four studies, which are methodologically similar and thus used a fixed-effect approach in which the mean effect size was weighted by sample size. At first, individual effect sizes were calculated separately for RTs and errors with the following formula (Cumming, 2014):

$$ {d}_{av}=\frac{M_{diff}}{\ {S}_{AV}\ }. $$

*M*_*diff*_ refers to the difference in emotional distraction between congruent and incongruent conditions. A positive value indicates that emotional distraction in incongruent conditions is smaller compared with congruent conditions. A negative value indicates that emotional distraction in congruent conditions is smaller compared to incongruent conditions.

$$ {M}_{diff}={M}_{EDcon}-{M}_{EDinc,} $$

*S*_*AV*_ refers to the pooled standard deviation of emotional distraction in congruent and incongruent conditions,

$$ {S}_{AV}=\sqrt{\frac{SD_{EDcon}^2+{SD}_{EDinc}^2}{2}}. $$

Then, *d*_*z*_ was corrected with Hedges’s method (Hedges, 1982):

$$ {g}_z={d}_z\times \left(1-\frac{3}{4\times \left(N-1\right)-1}\right), $$

in which N represents the sample size. The sampling variance (*v*_*i*_) was calculated according to Cumming (2014).

$$ {v}_i=\left(\frac{1}{N}+\frac{dz^2}{2N}\right)\times {\left(1-\frac{3}{4\times \left(N-1\right)-1}\right)}^2. $$

**Fig. 2:**
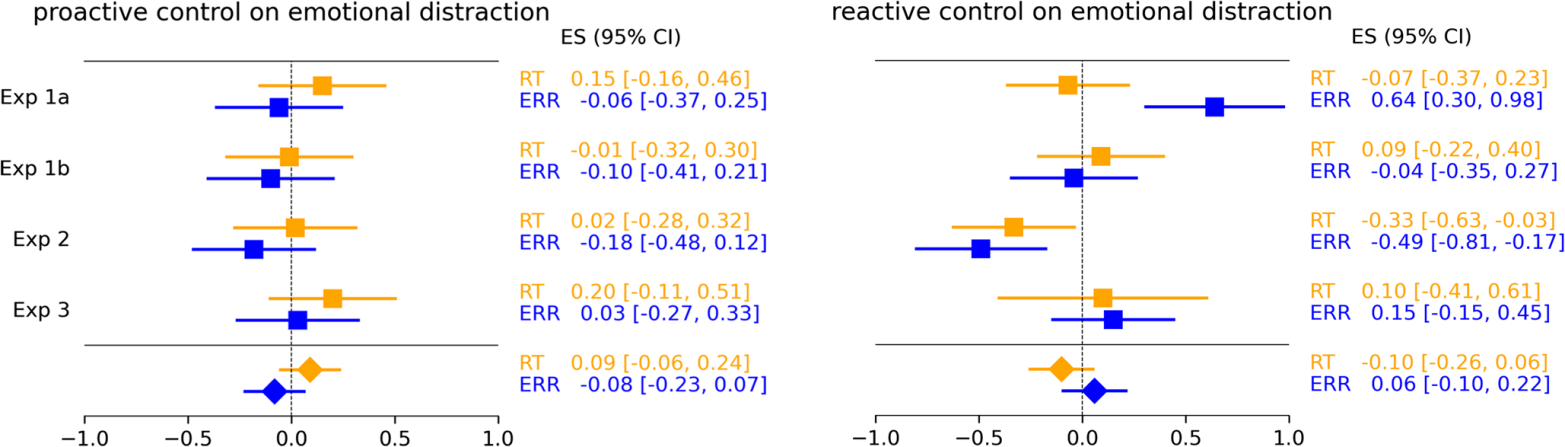
Effect size estimates of proactive reactive control on emotional distraction. *Note.* Observed effect size estimates of the difference of emotional distraction between congruency conditions in 'proactive control on emotional distraction' and 'reactive control on emotional distraction' in RT and error analysis of Experiments 1a,b,2,3 and the overall effect sizes (represented with a diamond) with their 95% confidence intervals

Mini meta-analyses were conducted separately for RTs and errors in R (R Core Team, 2015) using the metafor package (Viechtbauer, 2010). Results showed no effects of congruency on emotional distraction in 'proactive control on emotional distraction' and 'reactive control on emotional distraction'. Specifically, the estimate for 'proactive control on emotional distraction' in RTs (*M* = 0.09, 95% confidence interval [CI] = [−0.06, 0.24]) was statistically nonsignificant, z = 1.16, *p* = 0.25, nor was the estimate for 'reactive control on emotional distraction' for RTs (*M* = −0.10, 95% CI = [−0.26, 0.06]), z = −1.30, *p* = 0.19. Furthermore, the estimate for 'proactive control on emotional distraction' in error rates (*M* = −0.08, 95% CI = [−0.23, 0.07]) was statistically nonsignificant, z = −0.99, *p* = 0.32, nor was the estimate for 'reactive control on emotional distraction' for error rates (*M* = 0.06, 95% CI = [−0.10, 0.22]), z = 0.78, *p* = 0.44, see Figure 2 for individual and overall estimates for RTs and for error rates). The observation that all effect-size confidence intervals include the null strengthens our observation that conflict in Color Stroop tasks does not modulate Emotional Stroop effects.

## General Discussion

The study asked whether cognitive control (triggered in the Color Stroop task) attenuates emotional distraction in the Emotional Stroop task. This investigation was motivated by previous theoretical and empirical studies, suggesting that conflict-triggered activation of top-down monitoring processes suppress the effect of irrelevant emotional stimuli and thus attenuate emotional distraction (Cohen et al., 2012, 2015). We presented Color and Emotional Stroop tasks in an alternating-runs design and tested (i) how proactive control from the Color Stroop task modulates emotional distraction in the subsequent Emotional Stroop task ('proactive control on emotional distraction') and (ii) how reactive cognitive control from the Color Stroop task modulates emotional distraction that stems from the previous Emotional Stroop task and persists in time ('reactive control on emotional distraction'). We predicted that proactive and reactive cognitive control reduces emotional distraction. In three experiments (Experiments 1b, 2, 3) that manipulated predictability of congruency level (Experiment 2) and predictability of emotional content (Experiment 3), we found reliable congruency effects in Color Stroop tasks and emotional distraction within the Emotional Stroop task when emotional content was predictable. Moreover, in all three experiments, we also found a spillover of congruency effects and emotional distraction to the other task. More specifically, we observed prolonged responses in Emotional Stroop tasks following incongruent relative to congruent Color Stroop trials (i.e., conflict slowing effect, see Ullsperger et al., 2005; Verguts et al., 2011), and we observed prolonged responses in the Color Stroop tasks following negative relative to neutral Emotional Stroop trials, indicating that effects from both, incongruent Color Stroop trials, and negative Emotional Stroop trials cross task boundaries across trials to the other task.

Consistent with previous literature (McKenna & Sharma, 2004; Phaf & Kan, 2007), Emotional Stroop effects that were instigated by a previous Emotional Stroop task were more robust than Emotional Stroop effects within the Emotional Stroop task, which were mainly limited to conditions with the blockwise presentation of emotional words (Experiment 1b and 2). However, against our hypotheses, we did not find reliable modulations of emotional distraction by cognitive control. This absence of an interaction was supported by Bayesian evidence for the null-model “emotional distraction is not smaller in incongruent compared with congruent conditions” for 13 of 14 tests (BFs ranging from BF01 = 2.530 to BF01 = 15.357) and by a mini-meta-analysis across all reported experiments, suggesting that the confidence interval of the overall effect size for a modulation of emotional distraction by cognitive control includes the null. In the following, we will discuss the present results with regard to theoretical accounts and previous empirical findings.

### Implications for theories

A computational model of adaptive attentional control by Wyble et al. (2008) provides a detailed account of how cognitive control in Color and Emotional Stroop tasks interacts in terms of the conflict monitoring theory. It is assumed that a monitoring unit detects cognitive conflict and increases the activation level of a task demand unit, which represents the current task set. Consequently, processing of the task-relevant dimension is enhanced and distraction from the irrelevant task dimension in a consecutive trial is reduced. Wyble et al. (2008) propose that interference from task-irrelevant emotion stems from a “negative emotional node”, which exerts an inhibitory influence on current task representations (Wyble et al., 2008; see also Stolicyn et al., 2017 for a neurobiologically inspired model). According to their simulated data, cognitive control in incongruent Color Stroop trials suppresses the emotional node and reduces the impact of task-irrelevant emotional information on subsequent Emotional Stroop trials. In the present study, we provide an empirical test of this prediction. While we observed that (i) interference effects occur in incongruent Color Stroop tasks and emotional distraction occurs within Emotional Stroop tasks and (ii) effects operate across trials to other tasks (i.e., conflict slowing effects and emotional distraction in tasks subsequent to the Emotional Stroop task), our results do not support the hypothesis that emotional distraction instigated by Emotional Stroop tasks is modulated by cognitive control from Color Stroop tasks. This suggests that changes in attentional weights for the relevant dimension in Color Stroop trials following or during conflict do not affect how strongly the irrelevant dimension in the Emotional Stroop task (i.e., the meaning of the negative word) distracts performance. This limitation of cognitive control is incompatible with model simulations put forward by Wyble et al. (2008) and predictions that we derived from the model.

Furthermore, the observed limitation of cognitive control to block-off emotional distraction questions a domain-general view of cognitive and emotional control and suggests that cognitive control from Color Stroop tasks may not reflect the same conflict-triggered adaptation processes required to reduce emotional distraction. This may be the case even if both tasks share the same relevant and irrelevant dimension (but differ in terms of conflict instigated by cognitive tasks [competing response activation] and emotional tasks [general slow-down]). This dovetails with research that contrasts mechanisms involved in cognitive and emotional processes on behavioral and neural levels. These studies show that cognitive and emotional tasks are processed on different, domain-specific levels (Imbir et al., 2020; Kunde et al., 2012; Soutschek & Schubert, 2013) and are dissociable on a neural level (i.e., a lateral prefrontal cognitive control mechanism and a rostral anterior cingulate emotional control mechanism (Egner et al., 2008). Furthermore, our results indicate that Emotional Stroop tasks may not evoke cognitive control through the need for suppression of the emotional distraction (Okon-Singer et al., 2013), because this would potentially lead to an interaction with control from Color Stroop tasks (yet see an alternative interpretation of Vermeylen et al., 2020). The present results also might be of interest to the debate whether the Emotional Stroop effect is a special type of Stroop effect (Dalgleish, 2005) or is better characterized as a distinct phenomenon (Algom et al., 2004). Algom et al. (2004) present empirical data showing that Emotional Stroop effects behave differently than Color Stroop effects and argue in a conceptual analysis that the mechanisms underlying Color and Emotional Stroop tasks differ structurally and qualitatively. The results of the present research testing trial-by-trial combinations of Emotional and Color Stroop tasks show that these tasks do not interact, which strongly weights in favor of the independence of both tasks. It further indicates that Emotional Stroop tasks lack properties of Color Stroop tasks and vice versa and both effects are most likely distinct phenomena. Our results complement Algom et al.’s (2004) empirical observations and conceptual analysis by providing further evidence that Emotional and Color Stroop effects behave differently. Furthermore, by testing predictions that we derived from a computational model of the Emotional Stroop task, this research suggests that theoretical accounts of Emotional Stroop effects most likely require a cognitive architecture that differs from Cognitive Stroop models.

Finally, the present findings also are of interest for research on the scope of control in response-interference-tasks. It has been debated whether cognitive control in one task generalizes across trials to other tasks (see Braem et al., 2014 for a review). A critical boundary condition that has been put forward assumes that if two different tasks share the same relevant dimension, control acts across trials to other tasks (Notebaert & Verguts, 2008). The authors proposed that processing of relevant dimensions is enhanced in conflict trials, which improves processing of (the same) relevant dimension in another task. While the tasks in our study meet this criterion (i.e., Color and Emotional Stroop tasks vary the same relevant dimension) our results show no evidence for a generalization of cognitive control across trials. Speculatively, emotional tasks may represent an exception to this proposal; emotional stimuli distract the processing of a task’s relevant dimension in a conflict trial and thereby lever out any effects of cognitive control across tasks.

### Relation to previous research

The present study used different tasks and stimulus material to induce conflict and emotional distraction (i.e., Color and Emotional Stroop task) compared to previous research (i.e., Flanker or Simon tasks and emotional pictures, see Cohen et al., 2012, 2015). As outlined above, we chose these tasks to test predictions that we derived from the computational model of Wyble et al. (2008). This new combination of cognitive and emotional tasks showed that cognitive control from Color Stoop tasks does not modulate emotional distraction instigated by Emotional Stroop tasks. This observation contrast with previous research showing that under specific circumstances cognitive control seems to attenuate emotional distraction (e.g., Cohen et al., 2012, 2015). In the following, we speculate how differences in response-interference-tasks and control mechanisms, timing and emotional distraction could account for these differences.

First, the 4-choice Color Stroop tasks used in the present research differs from previous 2-choice flanker tasks in their complexity and thus resulted in different overall RTs (e.g., 4-choice Stroop: 700-1,000 ms vs. two-choice flanker: 400-800 ms, see Cohen et al., 2012, 2015; Straub et al., 2020). Many accounts hold that control takes time to develop suggesting that congruency effects differ between faster and slower responses (Ridderinkhof, 2002, see also Nieuwenhuis & de Kleijn, 2013 for the impact of alertness on cognitive control). Possibly, differences in overall RTs could explain the difference between the present and previous research. We tested this assumption in two post-hoc analyses that compared a possible interaction between cognitive control and emotional distraction across the RT distribution of each participant (within-subject comparison across percentiles) and averaged across all participants (between-subject comparison of relatively fast and slow participants). Both analyses replicated Color and Emotional Stroop effects but found no interaction between both, independent of the overall RT level. Thus, different overall RT levels do not viably explain the diverging results of our and previous studies.

Second, the type of conflict and consequently, conflict resolution mechanisms, differ between response-interference tasks. While semantic and response conflict (De Houwer, 2003), as well as task conflict (Goldfarb & Henik, 2007), contribute to Stroop interference, flanker tasks create stimulus, and response conflict (van Veen & Carter, 2005). Furthermore, in the Stroop task, which has been used in the present research, control is concerned with feature-based attention (color vs. word), whereas in flanker tasks used in most of the studies by Cohen and colleagues, control has been attributed to changes in spatial attention (Wendt et al., 2012). Regarding mechanisms of conflict resolution, it has been suggested that in the Stroop task control leads to an amplification of the task-relevant dimension (Egner et al., 2007; Egner & Hirsch, 2005), whereas control in the Simon task (e.g., used in combination with affect in Fruchtman-Steinbok et al., 2017) is usually described as an inhibition of automatic response activation by the irrelevant dimension (Stürmer et al., 2002). Although largely speculative, differences between tasks could account for heterogeneous findings whether cognitive control does or does not modulate affective processing.

Third, differences in emotional stimulus material and processing of emotional information could account for the discrepant results. In contrast to many previous studies that presented emotional pictures, the present research used emotional words, which have been criticized as ecologically invalid (Schimmack & Derryberry, 2005), and there has been a debate whether affective responses differ between words and pictures (Hinojosa et al., 2009; Kensinger & Schacter, 2006). Furthermore, it has been suggested that cognitive control over emotional distraction is limited to situations in which emotional stimuli are either task-relevant (i.e., when participants evaluated the valence of pictures) or entirely task-irrelevant (i.e., when participants ignored pictures completely). However, responding to a feature of the picture different than valence failed to produce an interaction between cognitive control and emotional distraction (see Cohen et al., 2016). Possibly, the lack of an interaction in the present study could be explained by the “implicit processing” account (see Cohen et al., 2016 for a detailed description of the account) of emotional stimuli (i.e., subjects respond to the print-color of the emotional words and not to the word’s emotional content). However, other research suggested that attention to emotional stimuli (but not task-relevance) is a necessary condition for emotional distraction ( Kanske, 2012; Okon-Singer et al., 2007) and cognitive control over emotional stimuli (Kanske & Kotz, 2011). Therefore, future research should test specific moderators of cognitive control - emotion interactions.

## Conclusions

Our experiments demonstrated that while Color and Emotional Stroop effects specific to one task impact on the other task, conflict-triggered control in the Color Stroop does not modulate emotional distraction at different timescales. This constrains theoretical accounts of control in cognitive and emotional tasks that predict such an interaction for tasks that recruit the same relevant dimension. Rather, the present results point out severe limitations of cognitive control to generalize across tasks when cognitive tasks were intermixed with emotional tasks.

## Appendix

**Table 1.** Words from Berlin affected word list (Võ et al., 2009)

WORD BAWL word list	Negative			WORD BAWL word list	Neutral		
	Valence	Arousal	Letter		Valence	Arousal	Letter
**Experiments 1a and 1b**							
TYRANN	-2.60	3.81	6	UHR	0.09	1.79	3
PEST	-2.80	4.00	4	DING	-0.03	1.56	4
VERGASEN	-2.80	4.00	8	LAGE	-0.06	1.83	4
QUAL	-2.70	4.11	4	BODEN	0.05	1.56	5
LYNCHEN	-2.60	4.21	7	EIMER	0.10	1.65	5
ANGST	-2.60	4.38	5	SILBE	0.15	1.72	5
GEWALT	-2.70	4.38	6	MATTE	0.15	1.72	5
MORDEN	-2.70	4.39	6	SOHLE	0.00	1.78	5
TÖTEN	-2.71	4.43	5	ANZAHL	0.20	1.71	6
FOLTER	-2.80	4.68	6	LOCHER	-0.14	1.82	6
TUMOR	-2.70	4.50	5	KARTON	0.00	1.84	6
TODFEIND	-2.70	4.53	8	DECKEL	-0.09	1.86	6
KRIEG	-2.90	4.57	5	BEREICH	0.03	1.78	7
MASSAKER	-2.80	4.61	8	SCHLICHT	0.00	1.67	8
FOLTERN	-2.80	4.68	6	MITGEBEN	0.10	1.78	8
TOD	-2.80	4.05	3	SCHRAUBE	0.00	1.86	8
ZERSTÖREN	-2.50	3.92	9	SCHWEIGEN	-0.26	1.73	9
WAFFE	-2.40	4.23	5	LINIE	0.10	1.84	5
LEBLOS	-2.30	3.50	6	QUADER	0.10	1.88	6
SUCHT	-2.30	4.00	5	TRAGEN	0.12	1.89	6
**Experiments 2-4** (4 words added)							
TYRANN	-2.6	3.81	6	UHR	0.090	1.79	3
PEST	-2.8	4.00	4	DING	-0.03	1.56	4
VERGASEN	-2.8	4.00	8	LAGE	-0.06	1.83	4
QUAL	-2.7	4.11	4	BODEN	0.05	1.56	5
LYNCHEN	-2.6	4.21	7	EIMER	0.10	1.65	5
ANGST	-2.6	4.38	5	SILBE	0.15	1.72	5
GEWALT	-2.7	4.38	6	MATTE	0.15	1.72	5
MORDEN	-2.7	4.39	6	SOHLE	0.00	1.78	5
TÖTEN	-2.7	4.43	5	ANZAHL	0.20	1.71	6
FOLTER	-2.8	4.68	6	LOCHER	-0.14	1.82	6
TUMOR	-2.7	4.50	5	KARTON	0.00	1.84	6
TODFEIND	-2.7	4.53	8	DECKEL	-0.09	1.86	6
KRIEG	-2.9	4.57	5	BEREICH	0.03	1.78	7
MASSAKER	-2.8	4.61	8	SCHLICHT	0.00	1.67	8
SEUCHE	-2.5	4.25	6	MITGEBEN	0.10	1.78	8
TOD	-2.8	4.05	3	SCHRAUBE	0.00	1.86	8
ZERSTÖREN	-2.5	3.92	9	SCHWEIGEN	-0.26	1.73	9
WAFFE	-2.4	4.23	5	LINIE	0.10	1.84	5
HASSEN	-2.5	4.40	6	QUADER	0.10	1.88	6
SUCHT	-2.3	4.00	5	TRAGEN	0.12	1.89	6
GIFTGAS	-3.0	4.20	7	VORKOMMEN	0.05	1.94	9
BLUTTAT	-2.8	4.28	7	ABSENDER	0.00	2.12	8
ALPTRAUM	-2.8	4.53	8	MANUELL	0.00	2.11	7
ATOMBOMBE	-2.8	4.42	9	STEMPEL	-0.05	2.00	7

**Table 2:** Interaction effects and main effects of Experiment 1-3

**Emotional distraction (ED) in congruent and incongruent conditions interaction effects congruency x valence**								
**RTs**								
	ED con proactive control		ED inc proactive control		ED con reactive control		ED inc reactive control	
	*M* [ms]	*SD* [ms]	*M* [ms]	*SD* [ms]	*M* [ms]	*SD* [ms]	*M* [ms]	*SD* [ms]
Exp1a	7	62	-3	68	18	73	23	82
Exp1b	18	58	19	49	18	72	13	42
Exp 2	14	42	15	39	13	43	28	50
Exp 3	6	42	-3	42	12	37	16	43
**Error Rates**								
	ED con proactive control		ED inc proactive control		ED con reactive control		ED inc reactive control	
	*M* [%]	*SD* [%]	*M* [%]	*SD* [%]	*M* [%]	*SD* [%]	[%]	*SD* [%]
Exp1a	0.26	3.77	0.47	2.93	8.55	23.53	-7.69	26.44
Exp1b	0.08	3.63	0.49	4.1	0.57	4.04	0.73	3.75
Exp 2	-0.02	3.86	0.58	2.77	0.10	3.41	2.07	4.34
Exp 3	-0.01	2.85	-0.1	2.94	0.80	3.2	0.31	3.1
**Mean RTs and Error Rates in neutral and negative conditions in proactive and reactive control on emotional distraction main effect valence**								
**RTs**								
	neutral proactive control		negative proactive control		neutral reactive control		negative reactive control	
	*M* [ms]	*SD* [ms]	*M* [ms]	*SD* [ms]	*M* [ms]	*SD* [ms]	*M* [ms]	*SD* [ms]
Exp1a	709	141	711	143	740	156	760	155
Exp1b	859	91	877	103	860	102	875	109
Exp 2	864	102	878	102	873	98	893	110
Exp 3	881	109	882	112	887	116	901	112
			**Error Rates**					
	neutral proactive control		negative proactive control		neutral reactive control		negative reactive control	
	*M* [%]	*SD* [%]	*M* [%]	*SD* [%]	*M* [%]	*SD* [%]	*M* [%]	*SD* [%]
Exp1a	4.13	2.7	4.47	3.18	9.38	12.66	10.26	12.16
Exp1b	7.33	3.66	7.61	3.52	7.32	3.22	7.97	3.8
Exp 2	7.09	3.87	7.37	4.58	7.3	3.92	8.38	4.56
Exp 3	7.67	3.71	7.61	4.43	7.38	4.42	7.93	4.54
**Mean RTs and Error Rates in congruent and incongruent conditions in proactive and reactive control on emotional distraction main effect congruency**								
**RTs**								
	congruent proactive control		incongruent proactive control		congruent reactive control		incongruent reactive control	
	*M* [ms]	*SD* [ms]	*M* [ms]	*SD* [ms]	*M* [ms]	*SD* [ms]	*M* [ms]	*SD* [ms]
Exp1a	711	144	709	141	683	139	817	178
Exp1b	860	91	876	103	806	91	929	122
Exp 2	864	103	878	103	818	102	948	109
Exp 3	873	112	890	110	831	111	957	121
**Error Rates**								
	congruent proactive control		incongruent proactive control		congruent reactive control		incongruent reactive control	
	*M* [%]	*SD* [%]	*M* [%]	*SD* [%]	*M* [%]	*SD* [%]	*M* [%]	*SD* [%]
Exp1a	4.55	3.07	4.05	2.84	9.83	13.22	10.26	14.61
Exp1b	7.34	4.16	7.59	3.11	6.61	2.76	8.68	4.24
Exp 2	7.14	3.73	7.32	4.64	6.46	3.64	9.22	5.15
Exp 3	7.5	3.77	7.78	4.46	6.51	3.75	8.81	5.64

## Footnotes

1 ERQ data of one participant were missing.

2 Alpha-corrected *p*-value = 0.0125

3 Corresponding to the minimum refresh rate (144 Hz) of the screen.

4 Note that *p*-value > 0.0125 were not significant according to the Bonferroni correction explained in the *Data Analysis* section.
